# Robotic Assisted Minimally Invasive Coronary Revascularisation: Midterm Results

**DOI:** 10.1002/rcs.70071

**Published:** 2025-05-05

**Authors:** Gökhan Arslanhan, Zeynep Sıla Özcan, Şahin Şenay, Murat Baştopçu, Anıl Karaağaç, Muharrem Koçyiğit, Aleks Değirmencioğlu, Deniz Alis, Cem Alhan

**Affiliations:** ^1^ Department of Cardiovascular Surgery Acibadem Mehmet Ali Aydınlar University School of Medicine Istanbul Turkey; ^2^ Department of Anesthesiology Acibadem Mehmet Ali Aydınlar University School of Medicine Istanbul Turkey; ^3^ Department of Cardiology Halic University School of Medicine Istanbul Turkey; ^4^ Department of Radiology Acibadem Mehmet Ali Aydınlar University School of Medicine Istanbul Turkey

**Keywords:** computed tomography guidance, coronary bypass surgery, coronary revascularisation, minimally invasive, robot‐assisted, robotic cardiac surgery

## Abstract

**Background:**

Robotic assistance has many advantages in minimally invasive coronary bypass surgery, such as the harvest of a longer portion of the LIMA in addition to the avoidance of sternotomy, thus offering a less invasive approach for multivessel revascularisation. We present the midterm clinical outcomes of robotic‐assisted minimally invasive coronary bypass (RA‐CABG) cases at our centre.

**Methods:**

One hundred and fifty consecutive patients who underwent RA‐CABG with preoperative computed tomography angiography guidance were studied. Robotic LIMA harvesting was performed. The main outcome measure of the study was the midterm survival and incidence of major adverse cardiovascular events (MACE) up to 5 years.

**Results:**

The median follow‐up was 19.8 months. In the Kaplan–Meier survival analysis, 1‐year survival was 99.1% and 5‐year survival was 97.5%. 1‐year freedom from MACE was 97.3% and 5‐year freedom from MACE was 95%.

**Conclusions:**

Robotic‐assisted minimally invasive coronary bypass surgery has safe midterm outcomes and can be performed with excellent results.

## 1 Introduction

Minimally invasive techniques are shown to be good alternatives to conventional CABG with sternotomy with good clinical outcomes when compared with traditional techniques [1, 2, 3]. Furthermore, robotic‐assisted minimally invasive coronary bypass surgery is the least invasive approach to surgical revascularisation in coronary artery disease. The use of robotic assistance in coronary bypass surgery may enable performing minimally invasive techniques that reduce the size of incisions, leading to less trauma and pain for patients with the elimination of the need for retractors, quicker recovery times and lower risks of complications with the avoidance of sternotomy. The enhanced visualisation and dexterity afforded by robotic technology also allow for more accurate and effective procedures, potentially improving overall surgical outcomes and patient prognoses.

However, clinical outcomes for this technique are not widely studied. We aim to share our midterm results with robotic‐assisted minimally invasive coronary revascularisation in this study.

## 2 Material and Method

### 2.1 Study Population

Between April 2010 and April 2024, 150 consecutive patients who underwent RA‐CABG were included. Preoperative computed tomography angiography (CTA) was performed for all patients and all cases were performed by a single surgical team with Da Vinci Robotic Systems. No RA‐CABG cases were excluded. Patients who presented with severe comorbidities, left pleural adhesions, emergent cases or patients with ascending aortic disease that prohibit robotic surgery did not undergo RA‐CABG. All other patients who underwent RA‐CABG with arrested heart or fibrillation technique, and off‐pump cases were included in the study. Pre‐operative patient demographics, perioperative and post‐operative data of the patients were retrospectively collected and analysed. The main outcome measures were midterm survival and the incidence of major adverse cardiovascular events (MACE) during 5‐year follow‐up. MACE was defined as coronary reintervention, cerebrovascular event or mortality.

### 2.2 Preoperative Planning With Computed Tomography Guidance

**FIGURE 1:**
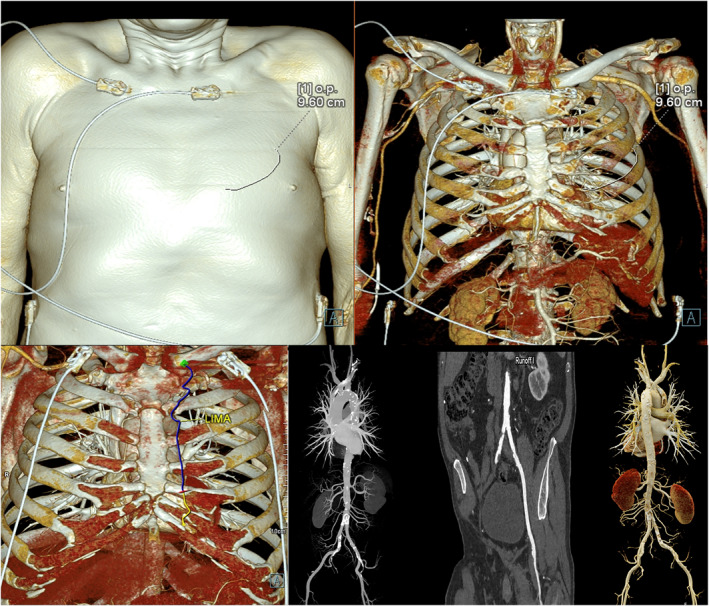
Computed tomography images of the thorax anatomy, LIMA topographic anatomy and peripheral arterial system and the aorta to guide pre‐operative planning.

It is our routine practice to perform CTA 5 days before the operation in all RA‐CABG patients. This enables us to preoperatively evaluate the anatomy of the thorax and the left internal mammary artery, angulations of the target diagonal artery from the LAD, the aorta, the minithoracotomy access site and any aortoiliac disease that would affect the choice of cannulation site (Figure 1). Comprehensive examination of the arteries was possible through the ECG gated CTA that was performed in retrospective spiral mode. Any tortuosity in the LIMA anatomy was taken into consideration to avoid damage to the artery during its harvest. Any thrombi, occlusion or calcifications in the iliofemoral system or the aorta were taken into consideration while making the decision for the cannulation site. The evaluation of any atherosclerosis of the ascending aorta was crucial in deciding on the strategy for proximal aortic anastomosis and on the choice of grafts. The decision for the thoracotomy access site was planned by assessing distances of the 3rd and 4th ICS to the ascending aorta at the level of the sinotubular junction and to the midportion of the LAD.

### 2.3 Surgical Technique

**FIGURE 2:**
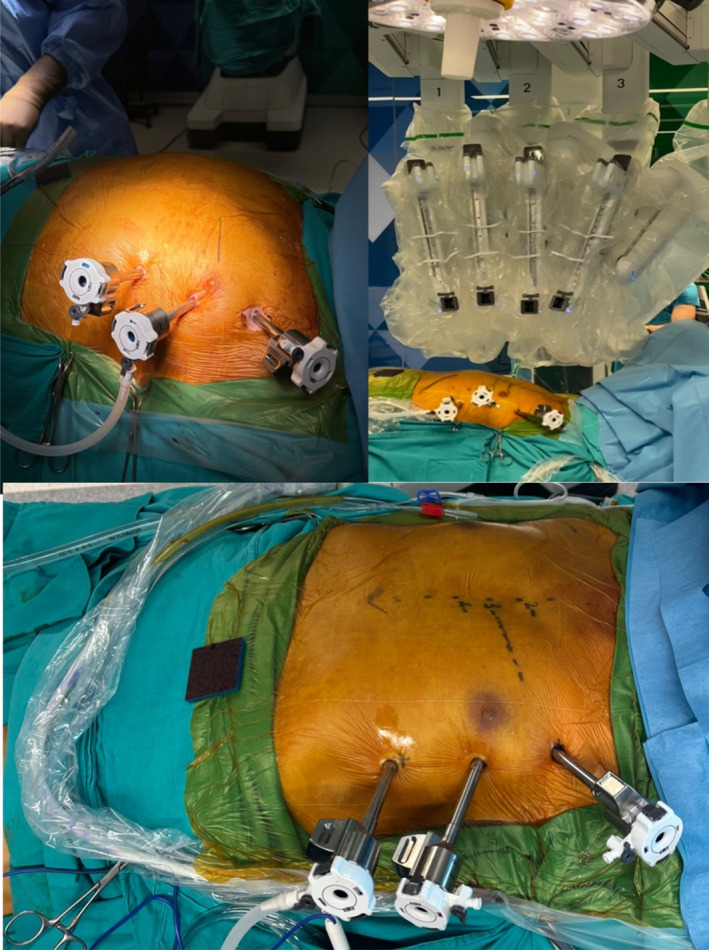
Skin markings and placement of the robotic ports.

**FIGURE 3:**
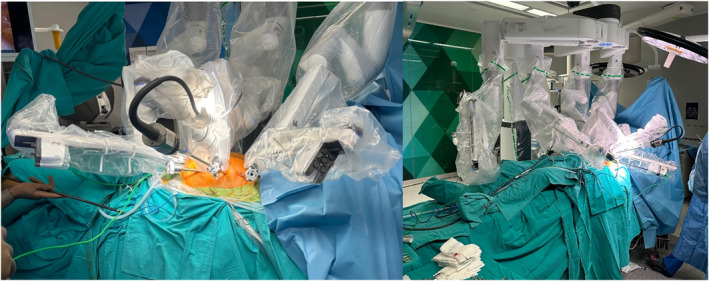
Robotic arms after the robotic docking is completed.

**FIGURE 4:**
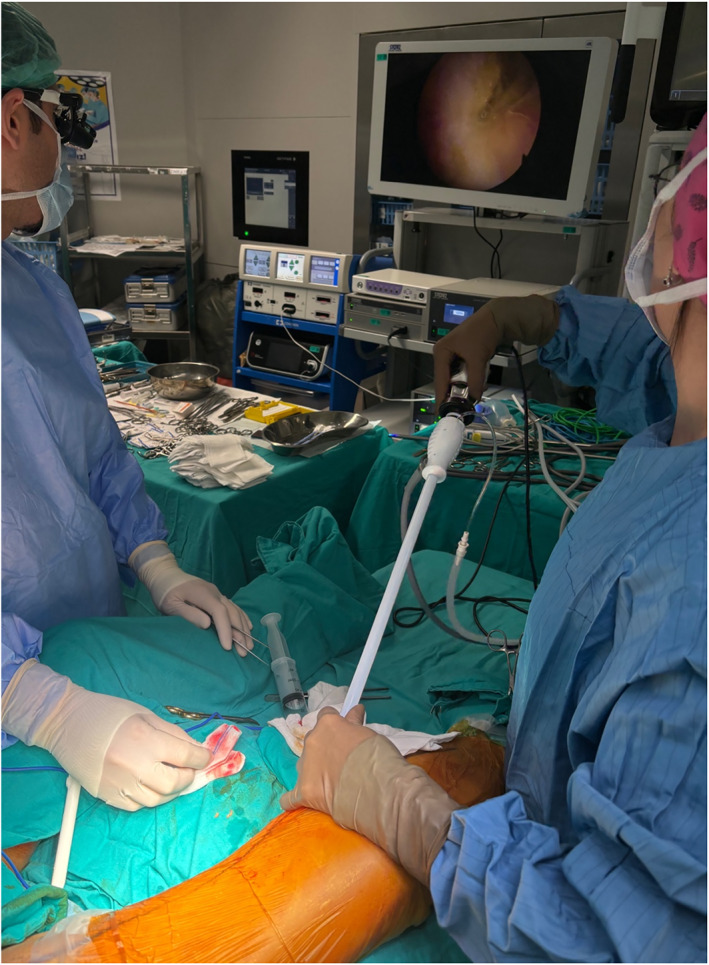
Endoscopic harvest of the saphenous vein.

**FIGURE 5:**
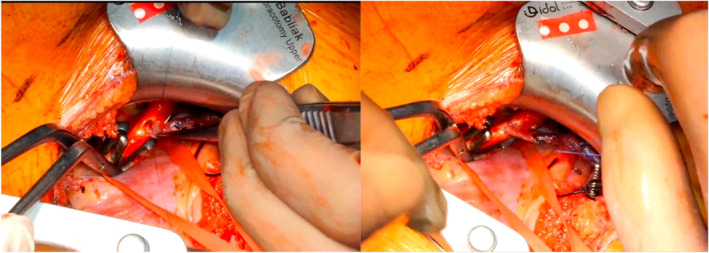
Performing the proximal anastomoses by positioning the heart and the aorta with the help of vascular tapes.

**FIGURE 6:**
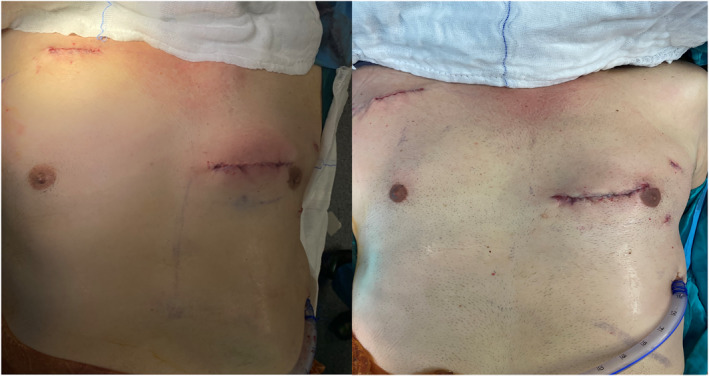
Thoracotomy and axillary cannulation incisions after skin closure.

Our surgical technique was described previously [4]. The patient is positioned for robotic setup, the induction of general anaesthesia is performed, a transesophageal echocardiography (TEE) probe was placed, and double‐lumen intubation was performed. According to the planning of the pre‐operative CTA, the skin markings for robotic trocar docking sites are made. Surgical draping was placed and the robotic trocars were then positioned. The right robotic trocar was placed through the second ICS, and this access site was later used for the insertion of the Chitwood clamp; the left robotic trocar was placed from the sixth or seventh ICS; and the camera was placed through the fourth ICS lateral to the nipple line (Figures 2 and 3). After the completion of robotic docking, the LIMA was harvested paying attention to specific variations in its course by taking into consideration the LIMA topography preoperatively visualised on the CTA. The dissection of the endothoracic fascia anterior and posterior to the left internal mammary artery was performed until the level of LIMA bifurcation was reached. Additional heparin with a target ACT above 250 was administered after the dissection of the entire length of the left internal mammary artery. Bleeding control of the thoracic wall was then performed through robotic assistance, and the robotic trocars were subsequently undocked. Anastomoses were performed with the robot in 10 TECAB cases among our 150 patients, and these were mostly single‐vessel patients. For the remaining patients, the LIMA was harvested robotically, and then the anastomoses were performed with long‐shaft minimally invasive instruments through the minithoracotomy. Axillary or femoral cannulation was planned according to preoperative CTA findings, and was performed under TEE guidance. The other surgeon simultaneously performed the thoracotomy incision. The Babliak minithoracotomy retractor was then placed. The heart was decompressed with the initiation of cardiopulmonary bypass (CPB) before opening the pericardium. The pericardium was incised, the pulmonary artery and the aorta were separated from each other and a Dacron tape was placed around the aorta using the Babliak technique [5]. The cardioplegia root cannula was placed, and the Chitwood clamp was then inserted through the second ICS, the access site previously used for the right robotic trocar. Cardioplegia was delivered and the heart was arrested. Distal exposure to the right side was achieved by encircling the left pulmonary veins and the inferior vena with tape and distal anastomoses were performed. The LIMA was grafted to the LAD in all patients, and LIMA was sequentially anastomosed to the diagonal artery if the angle between the LIMA and diagonal artery was smaller than 40° in the pre‐operative CTA evaluation. The radial artery was used when possible, and otherwise the saphenous vein was used as an additional graft in case of need. The additional grafts were endoscopically harvested (Figure 4). After the completion of the distal anastomoses, the cross clamp was removed and a side‐biting clamp was placed on the aorta to perform the proximal anastomoses (Figure 5). In some cases, proximal anastomoses were performed onto the LIMA. No proximal anastomoses were required in 2‐vessel cases in which the LIMA graft was anastomosed sequentially to the LAD and the diagonal artery. To ensure adequacy and quality of revascularisation, transit‐time flow measurements were performed after the completion of all anastomoses. The patient was weaned off CPB after the achievement of optimal conditions. A chest tube was then placed through the access site of the left robotic trocar, and all the other incisions were sutured (Figure 6). A temporary pacemaker was implanted only if needed.

### 2.4 Ethical Statement

The research was conducted with respect to the Declaration of Helsinki. Patient data were retrospectively retrieved from the institutional database. Approval was obtained from the Acıbadem Maslak Hospital Institutional Review Board before the establishment of the study (Maslak/15/06/2024). Consent was obtained from patients to include surgical images in the study.

### 2.5 Statistical Analysis

IBM SPSS Statistics, Version 25.0 (IBM Corp., Armonk, NY, USA) was used for statistical analysis. Continuous parameters are given as mean and standard deviation, while categorical parameters are given as numbers and percentages. Kaplan–Meier survival analysis was used for 1‐ and 5‐year survival and 1‐ and 5‐year freedom from major adverse cardiovascular events (MACE).

## 3 Results

Robotic‐assisted minimally invasive coronary bypass surgery under preoperative CTA guidance was performed on 150 consecutive patients between April 2010 and April 2024 at our institution. The mean age of the patients was 61.7 ± 9.6 years, and 122 patients were male (81%). 94 (63%) patients had pre‐existing hypertension, 69 (46%) patients had diabetes and 98 (65%) patients had hyperlipidaemia. The majority of the patients belonged to NHYA Class 2 with 75 (50%) patients. The mean EuroSCORE of the patients was 2.0 ± 1.8. The patient demographics are presented in Table 1.

**TABLE 1:** Preoperative characteristics of patients (*n* = 150).

Demographics	Mean ± SD or *n* (%)
Age (years)	61.7 ± 9.6
Female gender	28 (19%)
Euroscore II	2.0 ± 1.8
NYHA	
Class 1	70 (47%)
Class 2	75 (50%)
Class 3	5 (3.3%)
BMI	29.0 ± 5.9
Diabetes mellitus	69 (46%)
Hypertension	94 (63%)
Hypercholesterolaemia	98 (65%)
Smoking	
Never smoker	46 (31%)
Ex‐smoker	51 (34%)
Current smoker	53 (35%)

Abbreviations: BMI, body mass index; NYHA, New York Heart Association.

Among 150 patients, 52 (34.6%) were operated off‐pump. 10 patients among those operated off‐pump underwent robotic totally endoscopic coronary artery bypass surgery (TECAB). In the remaining 98 patients, axillary cannulation was the choice of perfusion in 61 (62%) patients due to prohibiting findings in the iliac vessels and aorta and femoral cannulation in 37 (38%) patients. Among the patients with aortic pathologies, 57% of the patients had severe calcification, 13% had thrombi and 30% had both. Based on CTA measurements, the left thoracotomy was performed through the 4th ICS in 88 patients (59%), through the 3rd ICS in 61 (41%) patients and through the 2nd ICS in one (0.7%) patient. All target coronary arteries with a bypass indication were effectively revascularised with CTA‐guided RA‐CABG. The mean number of revascularised vessels was 2.3 ± 1.2. The left internal mammary artery was anastomosed to the LAD in all patients. Radial artery and/or saphenous vein were preferred as a second graft if needed for the non‐LIMA grafts. Radial artery was preferred in young patients and left‐sided vessel anastomoses, and saphenous vein grafts were used when radial artery use was not possible. The diagonal artery was revascularised in 44 patients (29%), and sequential LIMA to LAD anastomosis was performed in 21 of these patients (14%). A radial artery graft was used in 19 patients (13%). The mean cross‐clamp times were 69.5 ± 20.3 min and cardiopulmonary bypass times 152.4 ± 43.5 min. The operative data are presented in Table 2.

**TABLE 2:** Operative data of RA‐CABG patients.

	Mean ± SD or *n* (%)
CPB time	152.4 ± 43.5
Cross clamp time	69.5 ± 20.3
Off‐pump cases	52 (34.6%)
On‐pump cases	98 (65.4%)
Axillary	61 (62%)
Femoral	37 (38%)
Thoracotomy incision	
2nd ICS	1 (0.7%)
3rd ICS	61 (41%)
4th ICS	88 (59%)
Number of distal anastomoses	
1	54 (36%)
2	30 (20%)
3	44 (29%)
4	15 (10%)
5	7 (4.7%)
Number of proximal anastomoses	
0	72 (48%)
1	59 (39%)
2	17 (11%)
3	2 (1.3%)
Usage of radial artery graft	19 (13%)
Lima sequential diagonal	21 (14%)

Abbreviations: CPB, cardiopulmonary bypass; ICS, intercostal space; LIMA, left internal mammary artery; RCAB, robotic coronary bypass; SD, standard deviation.

**FIGURE 7:**
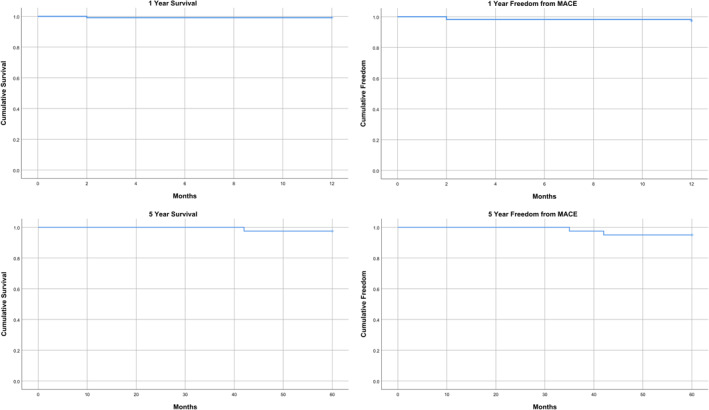
Kaplan–Meier analyses for 1‐year and 5‐year survival; 1‐year and 5‐year freedom from major adverse cardiovascular events (MACE).

The mean mechanical ventilation time was 4.9 ± 2.8 h. The mean intensive care unit stay was 20.2 ± 7.0 h and the mean hospital stay was 6.9 ± 2.4 days. The mean chest tube output was 429.1 ± 412.5 mL. The median follow‐up was 19.8 months. No early complications in the post‐operative 30‐day period were observed except in one patient who underwent reoperation due to bleeding. There was no operative mortality or cerebrovascular event. The postoperative outcome data are presented in Table 3. Late mortality was observed in 4 (2.7%) patients and 2 patients among them were lost due to non‐cardiac reasons. One of these patients was lost due to pulmonary emboli 2 months after the operation, one patient was lost due to cardiac arrest, and the other two patients were lost due to malignancy. Nine patients (6%) underwent late coronary reintervention. None of these patients were reoperated; all of them were revascularised with PCI. There were no cases of postoperative stroke; therefore, total MACE was observed in 13 (8.7%) patients. In the Kaplan–Meier survival analysis, 1‐year survival was 99.1% and 5‐year survival was 97.5%. The 1‐year freedom from major adverse cardiovascular events (MACE) was 97.3% and 5‐year freedom from MACE was 95% (Figure 7).

**TABLE 3:** Clinical outcomes.

	Mean ± SD or *n* (%)
Mechanical ventilation time (h)	4.9 ± 2.8
ICU stay (h)	20.2 ± 7.0
Hospital stay (days)	6.9 ± 2.4
Chest tube output (mL)	429.1 ± 412.5
Postoperative stroke (*n*)	0 (0%)
Postoperative dialysis (*n*)	0 (0%)
Reoperation for bleeding (*n*)	1 (0.006%)
Reintubation (*n*)	0 (0%)
Hospital readmission (*n*)	0 (0%)
Late coronary reintervention (*n*)	9 (6%)
Late mortality (*n*)	4 (2.7%)
MACE (*n*)	13 (8.7%)

Abbreviations: ICU, intensive care unit; MACE, major adverse cardiovascular events.

## 4 Discussion

Robotic‐assisted minimally invasive coronary revascularisation is a combination of two techniques which offers the advantages of both the robotic assistance and the minimally invasive technique in coronary bypass surgery. The short‐term results of this technique have been previously reported and are safe when compared to traditional coronary bypass surgical techniques [6]. The main indicator of this technique’s effectiveness is that the longer‐term results are also as good as those of conventional CABG. There is a paucity of data concerning midterm and long‐term results of robotic‐assisted multivessel CABG in the literature and other reports of robotic CABG include hybrid revascularisation cases, thus making a comparison and generalisation more difficult. In the limited number of reported series, 5‐year survival after RA‐CABG ranged between 82.7% and 86.8% [7, 8]. (Lo‐Torregrossa) In our series, we report our midterm results with 150 consecutive cases operated with robotic‐assisted minimally invasive coronary revascularisation. Our observance of a 1‐year survival of 99.1% and a 5‐year survival of 97.5% can be interpreted as RA‐CABG being an effective, feasible and safe technique for coronary revascularisation, providing many benefits for the patient and the surgeon. With growing experience, this technique can be performed with excellent results.

With the robotic‐assisted minimally invasive technique, sternotomy and its possible complications such as post‐operative sternal pain, sternal dehiscence and mediastinitis, wound infections, and an elongated post‐operative healing period are avoided [9]. Different studies analysing sternal healing after median sternotomy through combining findings of follow‐up computed tomography imaging and the clinic of patients have previously shown quite long sternal healing times ranging from 6 months to a year [10, 11, 12].

One of the concerns with the robotic‐assisted and minimally invasive technique in coronary bypass surgery is the quality and adequacy of the anastomoses. With robotic‐assistance, the LIMA can be harvested beyond the thoracotomy access and thus, 3rd or 4th ICS access for thoracotomy is feasible. The thoracotomy access site is planned according to the pre‐operative CTA after visualisation of the patient anatomy. In this way, easy and good exposure of the aorta is achieved without sacrificing the length of the harvested LIMA. As we more frequently started using the 3rd ICS for thoracotomy access in our more recent cases, the proximal and distal anastomoses can be easily completed with direct vision. The longer LIMA length also makes it more suitable for sequential anastomosis and side‐graft configurations [13]. Elimination of the need for any thoracic wall retraction during the LIMA harvest stage also allows for avoiding any nerve, muscle or rib injury. Full revascularisation of the coronary arteries is feasibly and comfortably achieved with this technique. The mean number of revascularised vessels was 2.3 ± 1.2 and up to 5‐vessel bypass surgery was performed in our study group. There was no need for the use of any further hybrid revascularisation techniques as all target vessels were surgically revascularised. We also routinely carry out transit‐time flow measurements to ensure perioperative anastomosis quality.

Another concern with minimally invasive cardiac procedures is the potentially increased rate of stroke in these patients, which is associated in some studies with the use of peripheral cannulation in minimally invasive access [14, 15, 16]. Coronary artery bypass patients present with a higher atherosclerotic burden compared with other patient groups. According to the pre‐operative CTA, we decide on the optimal cannulation site considering any aortoiliac atherosclerotic disease, free‐floating or mural thrombus, iliofemoral stenosis or soft plaque formation. If any of these conditions are present, we perform axillary cannulation instead of femoral cannulation. This helps reduce the risk of any cerebrovascular events, considering that a majority of coronary artery bypass patients also with peripheral atherosclerotic disease. We didn’t observe any cerebrovascular events in any of the patients in our study group and this was attributed to the elaborate preoperative investigation of the arterial system and an appropriate cannulation choice. With the routine use of preoperative CTA for operative planning, we aim to avoid the abovementioned potential complications of this technique with elaborate preoperative assessment of any calcification/thrombus in the peripheral arterial system and the aorta and the pre‐operative planning of the thoracotomy access site for easier completion of proximal anastomoses.

Our mean cardiopulmonary bypass times were 152.4 ± 43.5 min and mean cross‐clamp times were 69.5 ± 20.3 min. The learning curve plays an important role in mastering this technique. As we became more familiar with the technique, we achieved shorter cardiopulmonary bypass and cross‐clamp times. A well‐coordinated surgical team including surgeons, anaesthesiologists, scrub nurses and perfusionists is crucial in establishing a successful practice of robotic assisted minimally invasive coronary revascularisation programme. Procedural safety and clinical outcomes improve as the team gets exposed to more cases and becomes more familiar with all aspects of the procedure. In a study by Patrick et al., involving 1195 patients from the Society of Thoracic Surgeons Registry undergoing RA‐MIDCAB, it was reported that the learning curve is initially steep, but safer and more stable clinical outcomes are achieved after the 10th procedure [17]. In another analysis by Van den Eyde et al. on the first 300 RA‐CABG procedures performed at a single centre in Europe, procedural safety was observed to substantially improve after 50 cases [18]. This was interpreted as the safe and feasible integration of RA‐CABG into surgical programs being possible with complication rates quickly decreasing below the rates normally expected in traditional CABG.

In the Kaplan–Meier survival analysis, our 1‐year survival was 99.1% and 5‐year survival was 97.5%. 1‐year freedom from major adverse cardiovascular events (MACE) was 97.3% and 5‐year freedom from MACE was 95%. Our survival and freedom from MACE show that this technique is safe and feasible when compared with conventional CABG and non‐robotic minimally invasive CABG techniques. In a study of 607 cases undergoing conventional CABG procedure with median sternotomy, Kamel et al. reported an in‐hospital mortality of 7.4% and late mortality of 2.4%. The 2‐year freedom from MACE was 18.7% in this cohort [19]. In another study by Weintraub et al., including 3939 patients undergoing conventional CABG, the 1‐year survival was 97.6% and 5‐year survival 91.9% [20]. Hoffmann et al. reported their 1‐, 3‐ and 5‐year survival with direct minimally invasive CABG in high‐risk patients with multivessel disease as 77%, 62% and 48%, respectively [21]. Lin et al. reported lower in‐hospital and long‐term mortality with RA‐CABG compared with conventional CABG, with similar rates of target lesion or target vessel revascularisation, myocardial infarction, and stroke [22]. In another review by Bonatti et al. of the literature on long‐term results after robotically assisted coronary bypass surgery, 5‐year survival after RA‐CABG was higher than 90% and graft patency between 3 and 5 years was reported to be above 90% [23]. The 5‐year freedom from MACE rate was approximately 75% [23]. Our results also confirm that RA‐CABG can be safely performed using low mortality and complication rates. Taking all these studies and the outcomes of our study into consideration, the surgical outcomes of RA‐CABG are consistent and comparable with results published for open CABG through sternotomy.

There are certain limitations to our study. It was a non‐randomized retrospective observational study. Our results are from a single centre with a single group of operating surgeons. The number of included patients is limited, which makes it more difficult to generalise results. However, we report that good clinical outcomes can be achieved with RA‐CABG, which can lead to the consideration that this technique is safe and feasible for multivessel coronary revascularisation when compared with conventional CABG.

## 5 Conclusion

Robotic‐assisted minimally invasive coronary revascularisation has many advantages including avoidance of sternotomy, harvest of a longer LIMA, less aortic manipulation and more comfortable distal and proximal anastomosis. It has safe midterm outcomes and can be performed with excellent results. Complete target vessel revascularisation can be safely and feasibly achieved with this technique.

## Author Contributions

G.A., Z.S.Ö., M.B. and Ş.Ş. were responsible for the concept and design of the study and drafting of the article. All authors were involved in data collection, data interpretation, critical revision of the article and approval of the article.

## Ethics Statement

Ethical approval was obtained from the Acibadem Maslak Hospital Institutional Review Board.

## Conflicts of Interest

The authors declare no conflicts of interest.

## Permission to Reproduce Material From Other Sources

The authors have nothing to report.

## Data Availability

Data of the study will be made available upon request.

## References

[1] P. Rabindranauth , J. G. Burns , T. T. Vessey , M. A. Mathiason , K. J. Kallies , and V. Paramesh , “Minimally Invasive Coronary Artery Bypass Grafting Is Associated With Improved Clinical Outcomes,” Innovations 9 (2014): 421–426, 10.1177/155698451400900605. 25438111

[2] S. Al‐Ruzzeh , W. Mazrani , J. Wray , et al., “The Clinical Outcome and Quality of Life Following Minimally Invasive Direct Coronary Artery Bypass Surgery.” Journal of Cardiac Surgery 19, no. 1 (2004): 12–16, 10.1111/j.0886-0440.2004.04003.x. 15108783

[3] D. M. Holzhey , J. P. Cornely , A. J. Rastan , P. Davierwala , and F. W. Mohr , “Review of a 13‐Year Single‐ Center Experience With Minimally Invasive Direct Coronary Artery Bypass as the Primary Surgical Treatment of Coronary Artery Disease,” Heart Surgery Forum 15, no. 2 (2012): 61, 10.1532/hsf98.20111141. 22543338

[4] G. Arslanhan , Z. S. Özcan , Ş Şenay , et al., “Robot‐ Assisted Minimally Invasive Multivessel Coronary Bypass Guided by Computerized Tomography,” Innovations: Technology and Techniques in Cardiothoracic and Vascular Surgery 19, no. 1 (2023): 30–38, 10.1177/15569845231213038. 38111997

[5] O. Babliak , V. Demianenko , Y. Melnyk , K. Revenko , L. Pidgayna , and O. Stohov , “Complete Coronary Revascularization via Left Anterior Thoracotomy,” Innovations 14, no. 4 (2019): 330–341, 10.1177/1556984519849126. 31106625

[6] A. Dokollari , S. Sicouri , O. Erten , et al., “Long‐Term Clinical Outcomes of Robotic‐Assisted Surgical Coronary Artery Revascularisation,” EuroIntervention 20, no. 1 (2024): 45–55, 10.4244/eij-d-23-00373. 37994042 PMC10756223

[7] C.‐Y. Lo , C.‐L. Yu , Y. Chang , and H.‐J. Wei , “Long‐Term Results of Robotic‐Assisted Coronary Artery Bypass Grafting With Composite Arterial Grafts for Multiple Coronary Anastomoses: 10‐Year Experience,” Journal of Robotic Surgery (2022), 10.1007/s11701-022-01391-z. 35316487

[8] G. Torregrossa , M. P. Sá , J. Van Den Eynde , et al., “Robotic‐Assisted Versus Conventional Off‐Pump Coronary Surgery in Women: A Propensity‐Matched Study.” Journal of Cardiac Surgery 37, no. 11 (2022): 3525–3535, 10.1111/jocs.16878. 35998275

[9] A. M. Kleiman , D. T. Sanders , E. C. Nemergut , and J. L. Huffmyer , “Chronic Poststernotomy Pain,” Regional Anesthesia and Pain Medicine 42, no. 6 (2017): 698–708, 10.1097/aap.0000000000000663. 28937533

[10] B. Wang , D. He , M. Wang , et al., “Analysis of Sternal Healing After Median Sternotomy in Low Risk Patients at Midterm Follow‐Up: Retrospective Cohort Study From Two Centres,” Journal of Cardiothoracic Surgery 14, no. 1 (2019): 193, 10.1186/s13019-019-1000-1. 31711516 PMC6849321

[11] J. Raman , S. Lehmann , K. Zehr , et al., “Sternal Closure With Rigid Plate Fixation Versus Wire Closure: A Randomized Controlled Multicenter Trial,” Annals of Thoracic Surgery 94, no. 6 (2012): 1854–1861, 10.1016/j.athoracsur.2012.07.085. 23103010

[12] K. B. Allen , V. H. Thourani , Y. Naka , et al., “Randomized, Multicenter Trial Comparing Sternotomy Closure With Rigid Plate Fixation to Wire Cerclage.” Journal of Thoracic and Cardiovascular Surgery 153, no. 4 (2017): 888–896.e1, 10.1016/j.jtcvs.2016.10.093. 27923485

[13] U. Kappert , J. Schneider , R. Cichon , et al., “Wrist‐ Enhanced Instrumentation: Moving Toward Totally Endoscopic Coronary Artery Bypass Grafting,” Annals of Thoracic Surgery 70, no. 3 (2000): 1105–1108, 10.1016/s0003-4975(00)01801-4. 11016388

[14] E. Y. Chan , D. M. Lumbao , A. Iribarne , et al., “Evolution of Cannulation Techniques for Minimally Invasive Cardiac Surgery a 10‐Year Journey,” Innovations: Technology and Techniques in Cardiothoracic and Vascular Surgery 7, no. 1 (2012): 9–14, 10.1097/imi.0b013e318253369a. 22576030

[15] J. S. Gammie , Y. Zhao , E. D. Peterson , S. M. O’Brien , J. S. Rankin , and B. P. Griffith , “Less‐Invasive Mitral Valve Operations: Trends and Outcomes From the Society of Thoracic Surgeons Adult Cardiac Surgery Database,” Annals of Thoracic Surgery 90, no. 5 (2010): 1401–1410.e1, 10.1016/j.athoracsur.2010.05.055. 20971230

[16] M. Murzi , A. G. Cerillo , A. Miceli , et al., “Antegrade and Retrograde Arterial Perfusion Strategy in Minimally Invasive Mitral‐Valve Surgery: A Propensity Score Analysis on 1280 Patients,” European Journal of Cardio‐Thoracic Surgery 43, no. 6 (2013): e167–e172, 10.1093/ejcts/ezt043. 23404687

[17] W. L. Patrick , A. Iyengar , J. J. Han , et al., “The Learning Curve of Robotic Coronary Arterial Bypass Surgery: A Report From the STS Database,” Journal of Cardiac Surgery 36, no. 11 (2021): 4178–4186, 10.1111/jocs.15945. 34459029 PMC9128069

[18] J. Van Den Eynde , H. V. Bentein , T. Decaluwé , et al., “Safe Implementation of Robotic‐Assisted Minimally Invasive Direct Coronary Artery Bypass: Application of Learning Curves and Cumulative Sum Analysis,” Journal of Thoracic Disease 13, no. 7 (2021): 4260–4270, 10.21037/jtd-21-775. 34422354 PMC8339757

[19] A. T. H. Kamel , A. Hassouna , H. E.‐D. A. A. El‐Hamid , and T. S. Hikal , “Major Adverse Cardiac Events After First Time Elective Isolated Coronary Artery Bypass Grafting: A Retrospective Cohort Study.” Journal of the Egyptian Society of Cardio‐Thoracic Surgery 26, no. 4 (2018): 237–244, 10.1016/j.jescts.2018.11.001.

[20] W. S. Weintraub , S. D. Clements , L. V.‐T. Crisco , et al., “Twenty‐Year Survival After Coronary Artery Surgery,” Circulation 107, no. 9 (2003): 1271–1277, 10.1161/01.cir.0000053642.34528.d9. 12628947

[21] G. Hoffmann , C. Friedrich , K. Huenges , et al., “Minimally Invasive Direct Coronary Artery Bypass in High‐Risk Patients With Multivessel Disease,” Thoracic and Cardiovascular Surgeon 69, no. 7 (2021): 607–613, 10.1055/s-0041-1723845. 34044462

[22] T.‐H. Lin , C.‐W. Wang , C.‐H. Shen , et al., “Clinical Outcomes of Multivessel Coronary Artery Disease Patients Revascularized by Robot‐Assisted vs Conventional Standard Coronary Artery Bypass Graft Surgeries in Real‐World Practice,” Medicine 100, no. 3 (2021): e23830, 10.1097/md.0000000000023830. 33545949 PMC7837900

[23] J. Bonatti , J. Ramahi , F. Hasan , et al., “Long‐Term Results after Robotically Assisted Coronary Bypass Surgery,” Annals of Cardiothoracic Surgery 5, no. 6 (2016): 556–562, 10.21037/acs.2016.11.04. 27942487 PMC5135552

